# Hospital Meetings, &C.

**Published:** 1898-07-02

**Authors:** 


### HOSPITAL MEETINGS, &C.

### NATIONAL REFUGES FOR CHILDREN.

By invitation of the Committee of Management of the training ships, "Arethusa" and "Chichester," a large party visited the ships on Thursday in order to witness the distribution of prizes by Lord Jersey. His lordship was accompanied by Lady Beatrice Villiers, and there were also present Lady Hood of Avalon, Admiral Sir T. Dalrymple Hay, Sir John Branston, and Mr. Edward Rawlings. The visitors were greatly interested in the inspection of the boys, and Mr. Archibald E. Scott, chairman of the committee, paid a high tribute to the work accomplished by Captain Moore, for during the nine years that gentlemon had been in charge only one boy had run away out of the 1,600 he had had to deal with. The ships were established in 1866, and since that year about 6,500 boys have undergone training for the sea. Of this number 4,470 have joined the merchant service, 700 entered the Royal Navy, while about 900 have gone into the Royal Marines, the Army, and the Fire Brigade. The cost of each lad on board the ships was stated to be £25 per annum.

